# Automated assessment of the smoothness of retinal layers in optical coherence tomography images using a machine learning algorithm

**DOI:** 10.1186/s12880-023-00976-w

**Published:** 2023-02-02

**Authors:** Jamshid Saeidian, Tahereh Mahmoudi, Hamid Riazi-Esfahani, Zahra Montazeriani, Alireza Khodabande, Mohammad Zarei, Nazanin Ebrahimiadib, Behzad Jafari, Alireza Afzal Aghaei, Hossein Azimi, Elias Khalili Pour

**Affiliations:** 1 Faculty of Mathematical Sciences and Computer, Kharazmi University, No. 50, Taleghani Ave, Tehran, Iran; 2 Department of Medical Physics and Biomedical Engineering, Tehran University of Medical Sciences and Research Center for Science and Technology in Medicine, Tehran, Iran; 3 Retina Service, Farabi Eye Hospital, Tehran University of Medical Sciences, Tehran, Iran; 4 Department of Computer Sciences, Faculty of Mathematical Sciences, Shahid Beheshti University, Tehran, Iran

**Keywords:** Automated segmentation, Support vector regression, Inner plexiform layer, Outer plexiform Layer, Bland-Altman plot, Biomarker

## Abstract

Quantifying the smoothness of different layers of the retina can potentially be an important and practical biomarker in various pathologic conditions like diabetic retinopathy. The purpose of this study is to develop an automated machine learning algorithm which uses support vector regression method with wavelet kernel and automatically segments two hyperreflective retinal layers (inner plexiform layer (IPL) and outer plexiform layer (OPL)) in 50 optical coherence tomography (OCT) slabs and calculates the smoothness index (SI). The Bland–Altman plots, mean absolute error, root mean square error and signed error calculations revealed a modest discrepancy between the manual approach, used as the ground truth, and the corresponding automated segmentation of IPL/ OPL, as well as SI measurements in OCT slabs. It was concluded that the constructed algorithm may be employed as a reliable, rapid and convenient approach for segmenting IPL/OPL and calculating SI in the appropriate layers.

**Supplementary Information:**

The online version contains supplementary material available at 10.1186/s12880-023-00976-w.

## Introduction

Optical Coherence Tomography (OCT) is a non-invasive and powerful imaging technique that uses low-coherence interferometry to obtain high-resolution multidimensional images of biological retinal tissue [1]. OCT provides an in vivo cross-sectional image from the retinal layers which typically lose their normal characteristics, such as thickness, smoothness, and organization, as a result of various pathologic conditions, including the diabetic retinopathy, age-related macular degeneration (AMD), and retinal vascular events [2–5]. Thus, quantifying these structural changes of retinal layers by processing the OCT images will provide useful information about the severity of involvement and also response to treatments [6]. In this way, segmentation of retinal layers is essential for this purpose and has been proposed using manual, semi-automatic, and fully automated methods [7–11].

On spectral-domain OCT (SD-OCT), the healthy retina has an organized, layered structure that is highlighted by diverse reflectance patterns. As knowledge of SD-OCT increased, plenty of imaging biomarkers were suggested to estimate the visual prognosis, such as the amount of the central retinal thickness (which reflects the entire retina), the attenuation of the ellipsoid zone (EZ) or external limiting membrane (ELM) (which reflect the outer retinal incompetence) and disorganization of the retinal inner layers (DRIL) (which reflects the inner retina) [12–14]. Improved risk stratification utilizing new OCT biomarkers would aid in disease morphology characterization, advise the optimum use of specific therapies (e.g., anti-vascular endothelial growth factor (VEGF) therapy), enhance prognosis counseling, and help define the inclusion criteria for therapeutic trials more precisely [15].

Moreover, the inner plexiform layer (IPL; also known as the inner synaptic layer) is made up of synaptic connections between bipolar cell axons and ganglion cell dendrites. In the visual pathway, the IPL includes the synapse between second-order and third-order neurons. The outer plexiform layer (OPL; also known as the outer synaptic layer) made up synapses between photoreceptor cells and cells from the inner nuclear layer. Because ischemic processes and structural abnormalities such as epiretinal membranes (ERM), may possibly impact the inner portions of the retina, the research of biomarkers that particularly evaluate the inner retina has gained a lot of interest in recent years [16]. For instance, disorganization of the retinal inner layers (DRIL) is a newly discovered and reliably prognostic imaging biomarker. DRIL is defined by disorganization of inner retinal laminations as well as an inability to distinguish the borders between the layers. Sun et al. defined DRIL and established a connection between it and visual acuity (VA) in patients with diabetic macular edema (DME) for the first time [17]. Since then, DRIL has been implicated in a variety of retinal pathologies, including retinal vein occlusions (RVO), nonproliferative diabetic retinopathy (NPDR) and proliferative diabetic retinopathy (PDR) with or without DME, epiretinal membranes (ERM), uveitic cystoid macular edema (CME), central retinal artery occlusion, and macular telangiectasia [17–28]. While DRIL may be an excellent VA correlate in a variety of retinal ischemic disorders, precise and repeatable measurements of DRIL may be challenging. Interobserver agreement for DRIL assessment was low in individuals with macular edema due to RVO. Macular edema may obscure the boundaries necessary for an appropriate assessment of DRIL. So, prior to regular use of this measure in clinical practice, an automated and algorithmic surrogate of DRIL is likely to be needed [29].

In this regard, Cho et al. compared the correlations between the inner-retinal irregularity index, defined as the length ratio between the inner plexiform layer and retinal pigment epithelium, and visual outcomes before and after epiretinal membrane (ERM) surgery. The inner-retinal irregularity index was shown to be strongly associated with visual results before and after ERM surgery. They showed that this index has the potential to serve as a novel surrogate measure for inner-retinal damage as well as a predictive prognostic sign in ERM [30]. Neuron J, a semi-automated layer-tracing software program that is a plug-in module for ImageJ, a free JAVA-based image analysis software package, was used to compute the irregularity index. However, the software fails to identify the right route in certain places with low-contrast layers. In this instance, the tracing mode is switched to manual tracing mode in order to precisely measure the length of interest [30].

Since the manual segmentation using applications such as ImageJ is time-consuming and extremely vulnerable to intra- and interobserver variability, automated techniques have been developed to overcome these issues [31–35]. Researchers have long studied the thickness of different layers of the retina as an important and practical biomarker in the diagnosis and treatment of various retinal diseases. Numerous studies focused on segmenting the retinal layers in OCT images to generate retinal thickness maps as well as establishing a correlation between the produced maps and associated retinopathies [30, 36, 37]. The formation of the retina as a series of parallel layers stacked on top of one another has prompted researchers to consider the degree of irregularity and thickness of these layers in various retinal conditions [17–19, 27–29]. We previously introduced a novel biomarker -iris smoothness index (SI)-into anterior segment optical coherence tomography (AS-OCT) images of patients with Fuchs uveitis and developed an automated method for its measurement [38, 39]. The manual segmentation of retinal layers in OCT images using ImageJ software, which is time-consuming and needs a skilled operator, may restrict the practical applicability of SI quantification in retinal layers. To overcome these limitations, in the current study we aimed to develop an automated algorithm for calculating the SI in two hyperreflective retinal layers (inner plexiform layer (IPL) and outer plexiform layer (OPL)) in patients with varying stages of diabetic retinopathy and compare the results to those obtained by experts using the manual method as the ground truth.

## Methods

### Data acquisition and adjustment

The dataset for this study was supplied by the Farabi Eye Hospital at Tehran University of Medical Sciences in Tehran, Iran. The RTVue XR 100 Avanti device (Optovue, Inc., Fremont, CA, USA) was used to capture enhanced-depth imaging optical coherence tomography (EDI-OCT) images between 8:00 and 12:00 a.m. Patients were positioned appropriately, and 8 mm * 12 mm raster patterns of OCT B-scans were captured. For image analysis and SI calculation, scans passing through the fovea as well as equally spaced rasters in the superior and inferior macular areas were chosen. In OCT scans above or below the fovea, the IPL and OPL layers are often separated and parallel. But, due to the near proximity of IPL and OPL in the foveal region, it might be challenging for the algorithm to differentiate between the layers.

To evaluate the accuracy of the algorithm in a robust and precise manner in the current investigation, we selected similar OCT rasters in all patients with equal distances from the fovea and assessed the accuracy of segmentation and measurement.

We omitted low-quality scans due to conditions like cataract or other causes of hazy media in which the IPL and OPL were not discernible. Due to structural OCT alterations, such as the presence of exudate, which may impact OPL and IPL segmentation, we also removed some OCT scans.

Finally, we included 50 OCT B-scans from 21 patients with various stages of diabetic retinopathy (mild to severe non-proliferative diabetic retinopathy and proliferative diabetic retinopathy) in this study. The images were originally 256 × 728 pixels in size. The inner plexiform layer (IPL) and outer plexiform layer (OPL) in OCT images were manually segmented by two retina experts (H.R.E and E.K.P using the public domain ImageJ software (available in the public domain at Fiji http://imagej.nih.gov/ijwas).

**Fig. 1:**
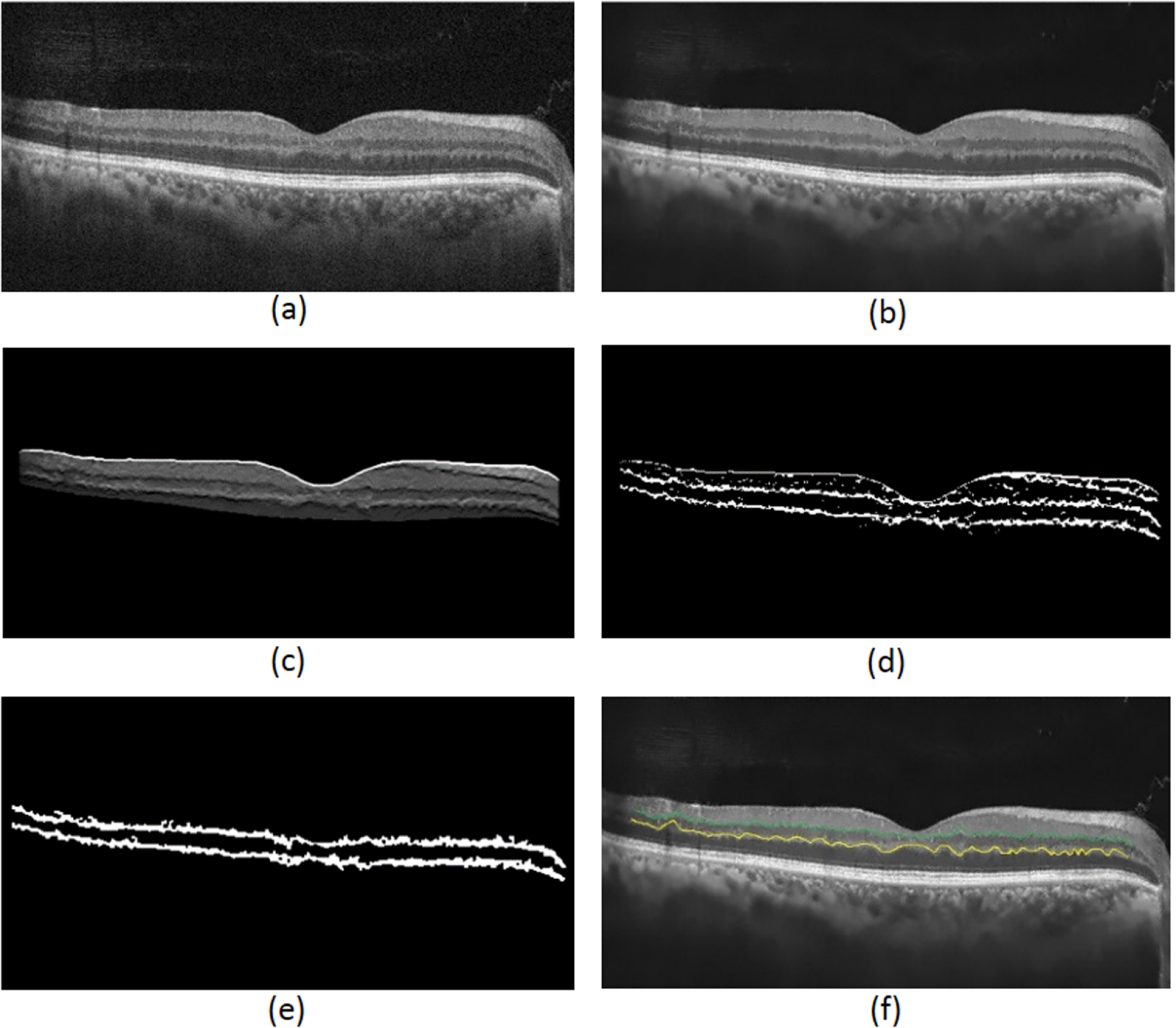
Steps of the proposed automated method. After denoising the original image (**a**) by non-local means denoising algorithm (**b**), the retinal area is localized using Gabor filters (**c**). A search strategy is used to perform the initial prediction of the IPL and OPL layers (**d**). Then dilation and erosion are performed in order to remove undesirable areas and improve the predicted layers (**e**). Finally, the boundaries are detected using an SVR-based model (**f**)

The proposed automated algorithm, developed in the current study by Python framework, consists of three stages: pre-processing; segmentation of the retinal IPL and OPL layers using support vector regression (SVR); and smoothness index calculation. The proposed steps of the automated method are depicted in Fig. 1. After denoising the original image (Fig. 1a) by non-local means denoising algorithm (Fig. 1b), the retinal area is localized using Gabor filters (Fig. 1c). A search strategy is used to perform the initial prediction of the IPL and OPL layers (Fig. 1d). Then, as morphological operations, dilation and erosion are performed in order to remove undesirable areas and improve the predicted layers (Fig. 1e). Finally, the boundaries are detected using an SVR-based model (Fig. 1f).

In preprocessing stage, low pass Gaussian filter with kernel size of 11 × 11 as well as a non-local means algorithm were used to reduce the noise of OCT images. This algorithm is an image noise reduction method that, unlike local and global methods, uses a different and smarter approach for denoising in such a way that it considers similar parts in image in terms of texture and structure, and detects and removes noisy points by averaging. Then, histogram equalization was applied to increase the contrast of the image.

**Fig. 2:**
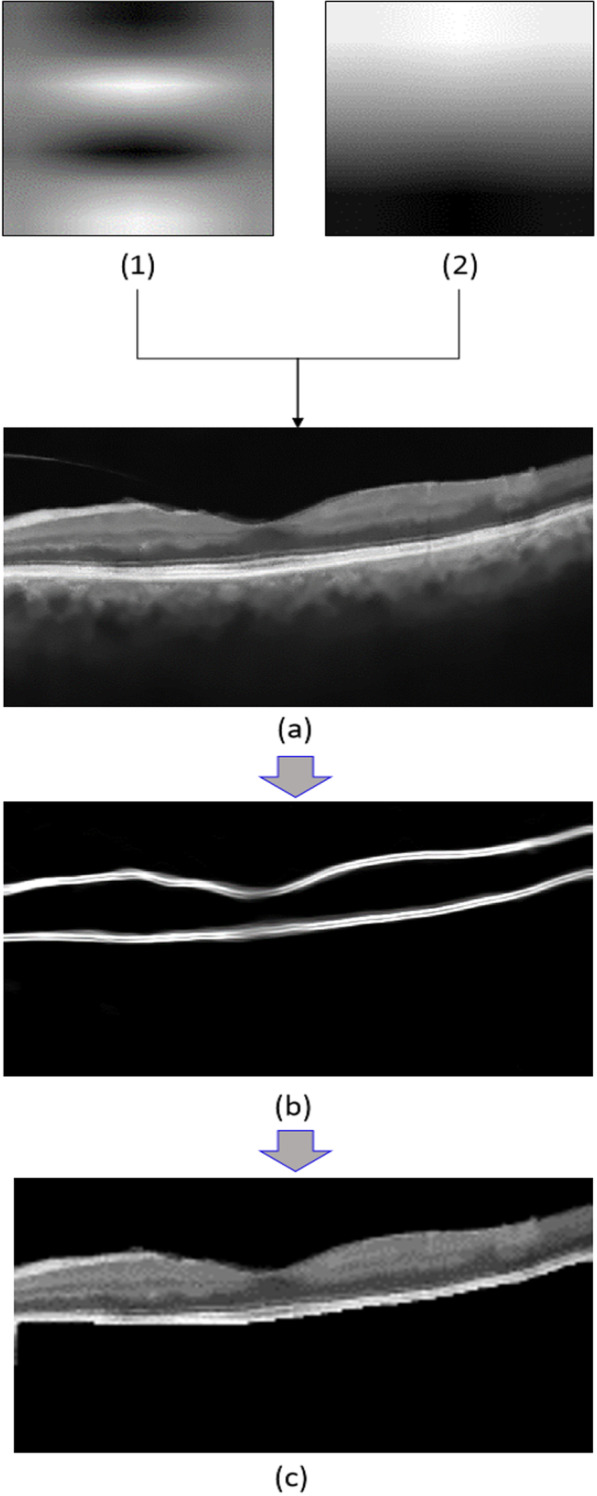
The result of localization of retinal area: (1,2) Gabor kernels, (**a**) Original image, (**b**) Upper and lower boundaries of the retina, (**c**) Segmented retina region

Localization of retinal area was carried out using Gabor filter. Upper and lower limits of the retina were obtained using segmentation of internal limiting membrane (ILM) and retinal pigment epithelium (RPE) layers. Gabor filtering with two different kernels was used for obtaining these boundaries as shown in the following formula.

1 $$g(x,y;\lambda ,\theta ,\psi ,\sigma ,\gamma ) = \exp \left( { - \frac{{x^{{\prime}{2}} + \gamma^{2} y^{{\prime}{2}} }}{{2\sigma^{2} }}} \right)\cos (2\pi \frac{{x^{\prime}}}{\lambda } + \psi ),$$

where

2 $${x}^{^{\prime}}=x\mathrm{cos}\theta +y\mathrm{sin}\theta ,{ y}^{^{\prime}}=-x\mathrm{sin}\theta +y\mathrm{cos}\theta ,$$

and $\lambda$ is the wavelength of sinusoidal component, $\theta$ represents the orientation of the normal to the parallel stripes of Gabor function, $\psi$ denotes the phase offset of the sinusoidal function, $\sigma$ is the standard deviation of the Gaussian envelope and $\gamma$ is the spatial aspect ratio and specifies the elasticity support of Gabor function. By properly selecting of $\lambda ,\theta ,\psi ,\sigma$ and $\gamma$ parameters, we obtained two different kernels that shown in Fig. 2(1,2). After applying them to the original image (Fig. 2a), the boundaries of the ILM and RPE were obtained that shown in Fig. 2b. In this way, as you can see in Fig. 2c the retinal area is separated from the rest of the image. The selected parameters for two kernels were summarized in Table 1. In the current study, the initial parameters were chosen in a way that the resulted segmentation visually resembles the output of the original OCT image. In this manner, we find a ground truth interval for the parameters. In the next step the values were carefully changed to increase the precision of the obtained layer according to the original image.

**Table 1:** Parameters used for two kernels for segmentation of ILM and RPE layers

Kernel	$\lambda$	$\theta$	$\psi$	$\sigma$	$\gamma$
Kernel 1 for ILM layer	$\frac{\pi }{4}$	$\frac{2\pi }{4}$	11	23	14
Kernel 2 for RPE layer	$\frac{\pi }{4}$	$\frac{2\pi }{4}$	− 1.5	20	11

### IPL and OPL segmentation

**Fig. 3:**
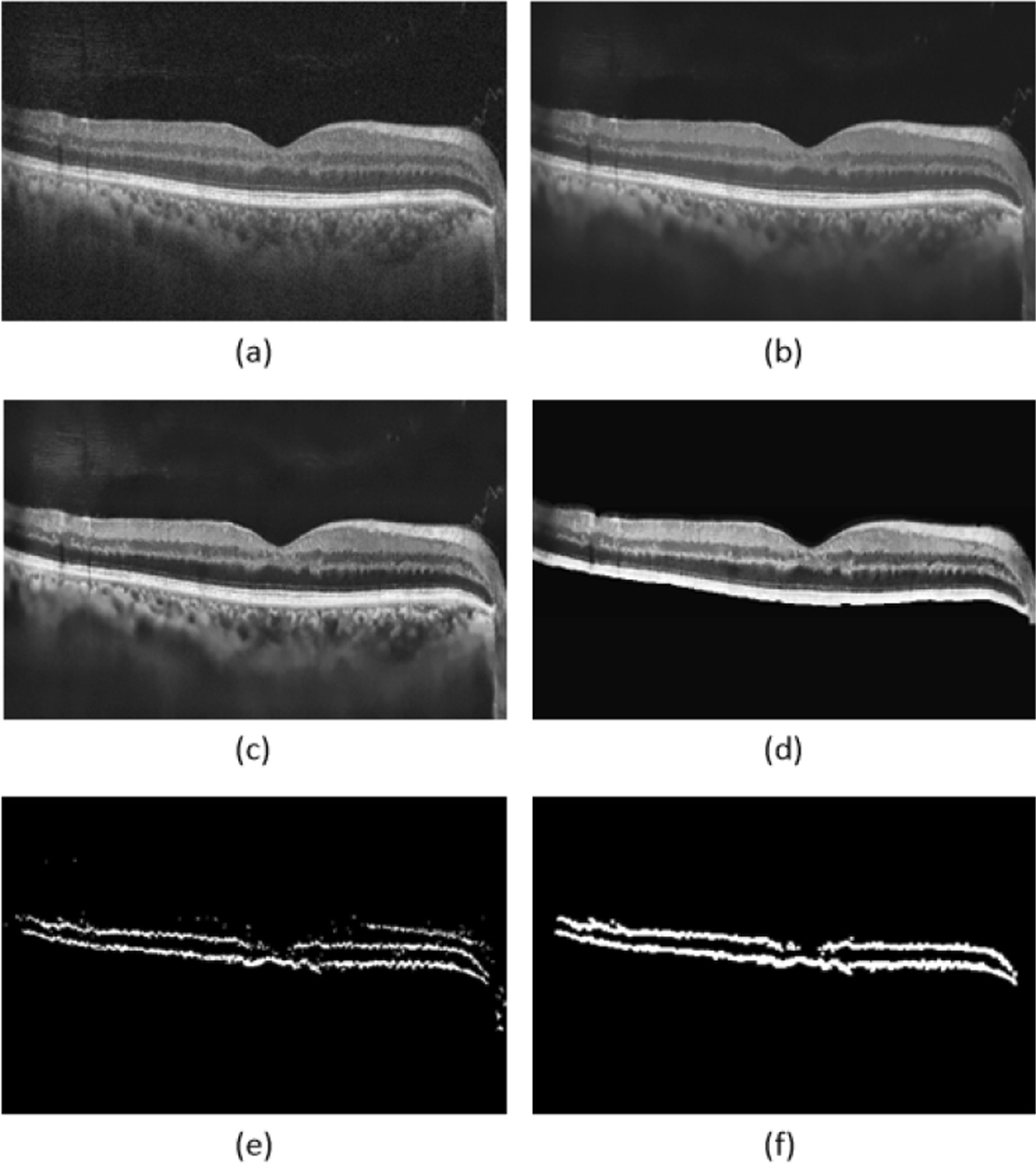
Pre-processing stage: **a** Original image, **b** Smoothed image using Gaussian filter, **c** Denoised image using non-localization method, **d** Enhanced image using histogram equalization, **e** Binarized image using a search strategy, **f** Result of applying morphological operators

We automatically acquired the retinal area as a region of interest in the previous steps, which has the benefit of reducing background noise and therefore facilitating the segmentation of the IPL and OPL layers. For segmentation of IPL and OPL layers, we smooth our original image (Fig. 3a) using Gaussian filter (Fig. 3b) and then go to reduce image noise (Fig. 3c). In the next step, we go to the enhanced image using histogram equalization that result of this process is shown in Fig. 3d. Initial prediction of the layers is obtained by employing a search strategy based on similarity and correlation between intensity of the pixels in these layers. According to the separability of the image histogram, after some mentioned preprocessing steps, conventional thresholding algorithms can be used to find the optimal threshold in a simple and achievable manner. The points on our target layers (IPL and OPL) have a brightness above 30, while other points reflect a brightness below 15, as can be observed after applying the Gabor filter. Testing different brightness thresholds between 15 and 30, one observes that the output images have no significant difference, so the threshold of brightness is set to 20 in the current study. After setting a suitable threshold between foreground (IPL and OPL) and background (the rest of the image) pixels, a binary image was created using global thresholding algorithm, this process is generally shown in Fig. 0.3. White pixels indicate estimated IPL and OPL locations in the generated binary images (Fig. 3e). Then, morphological operators are employed to detect the desired layers, erosion eliminates the individual white pixels which is not connected to any meaningful layer and dilation helps to get a connected and continuous layer. Also, we need to perform a connected-component labeling for eliminating any extra layer, such layers may occur in the process like the one which appears in the upper right corner of Fig. 3e. To be more precise, the process begins with two times erosion, followed by a dilation. Then it uses a connected component operator followed by an erosion and a dilation. The continuous boundaries were created and outliers were removed as depicted in Fig. 3f [40]. In order to skeletonize and get a curve-shape for IPL and OPL identifiers, we create two separate lists of pixels, the first is according to the far most upper white pixels in a column considered as IPL and the second stores the lowest white pixels in each column considered as OPL. It is possible for these two lists to have common points, it occurs whenever the IPL and OPL stick together in the original OCT slabs, in these cases this common pixels appear in both IPL and OPL lists, separately.

In the final step, due to discontinuity of pixels in the resulted lists, we need approximation methods for filling the gaps, that we will describe in the following sections.

**Fig. 4:**
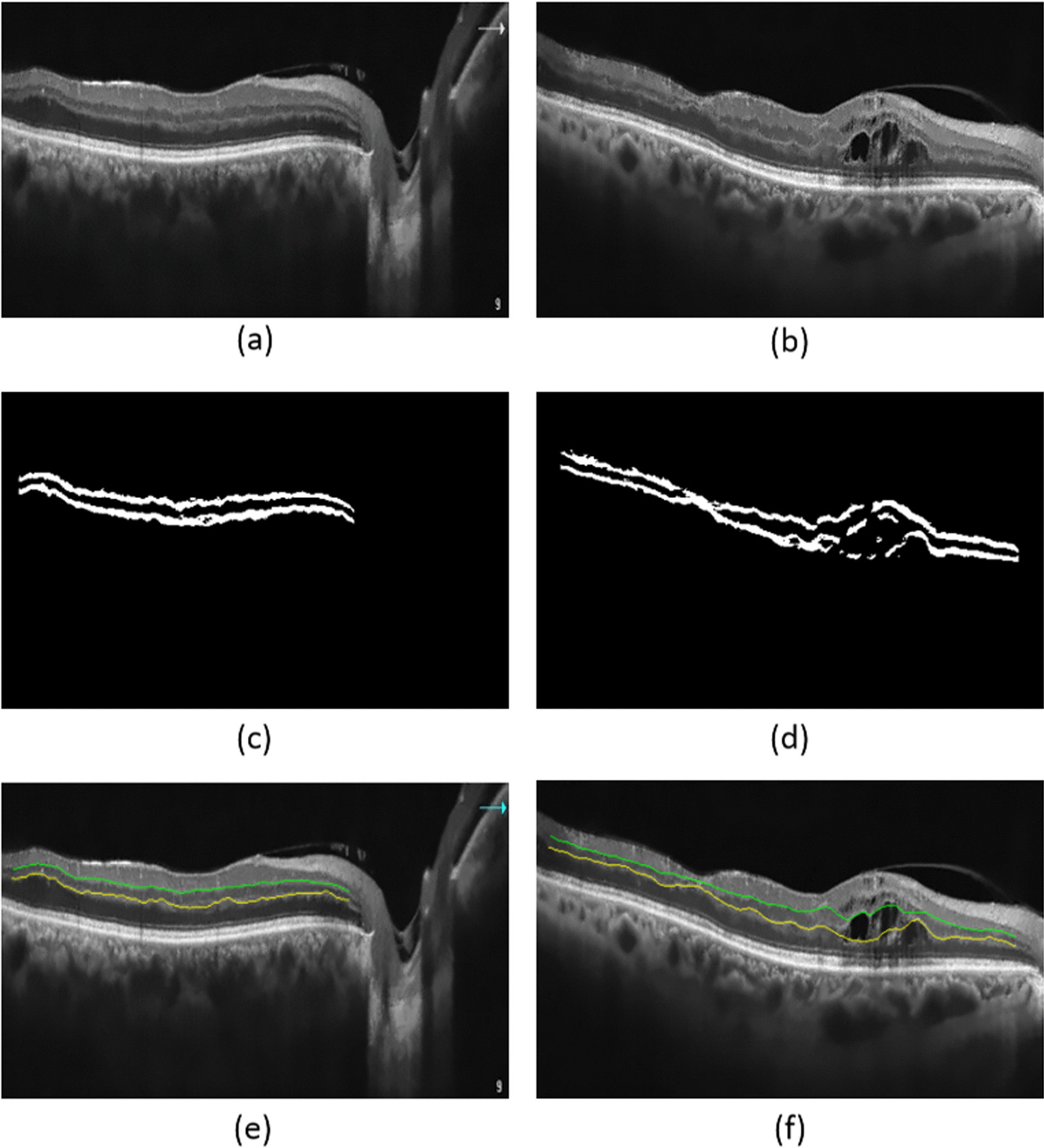
Segmentation results using cubic spline interpolation. **a** An OCT slab with relatively regular and organized layers. **c** The labelling of IPL and OPL for **a**, **e** Final segmentation for **a**, and **b** an OCT slab with disorganized layers, **d** the labelling of IPL and OPL for **b**, and **f** Final segmentation of the image **b** which shows incorrect segmentation of IPL

A modeling-based technique was utilized to achieve precise segmentation of the IPL and OPL layers. Two approaches were investigated for this purpose: classic and machine learning-based methods. In the classic technique, cubic spline interpolation is employed to determine the exact target layers’ (IPL and OPL) locations. Cubic spline interpolation is a method for modeling objects with curved shapes [41]. On OCT slabs with relatively regular and organized layers, this approach produces satisfactory results (Fig. 4c). However, as seen in Fig. 4, it fails in OCT slabs with relatively disorganized layers (Fig. 4f).

To address this issue, we presented a machine learning technique based on support vector regression (SVR). SVR, as a supervised-learning technique, works utilizing points on layers and a prediction function. When the labelling is wrong, the model predicts and replaces the missing values, which is an advantage of employing SVR.

Since our model was a nonlinear function, the data was transformed into a higher-dimensional space known as kernel [42]. SVR can be achieved by using various kernels such as polynomials [43], sigmoid [44], Radial Basis Functions (RBF) [45], and wavelet functions. The performance of SVR largely depends on the selection of the kernel. One of the interesting features of the kernel-based approach is its ability to use feature mapping with infinite dimensions. For this purpose, a class of functions known as radial basis functions (RBF) can be used [46] as follows:

3 $$K\left( {\varvec{x}.\varvec{x}^{\prime } } \right) = \exp \left( { - \frac{{\left\| {\varvec{x} - {\text{ }}\varvec{x}^{\prime } } \right\|^{2} }}{{2\sigma ^{2} }}} \right),$$

In the above equation, ${\varvec{x}}$ and ${{\varvec{x}}}^{^{\prime}}\in {\mathbb{R}}^{N}$, where ${\mathbb{R}}^{N}$ represents input space and $\sigma$ is the RBF kernel parameter. After mapping the data to an infinite-dimensional space, the SVR tries to fit a hyperplane in the feature space to the training data. So, our prediction function is mapped as Eq. (4):

4 $$y\left( {\varvec{x}} \right) = \mathop \sum \limits_{k = 1}^{n} \left( {a_{k}^{ + } - a_{k}^{ - } } \right) K\left( {{\varvec{x}}.\user2{x^{\prime}}_{{\varvec{k}}} } \right) + b,$$

where $y\in {\mathbb{R}}$ and we seek to estimate (4) based on independent uniformly distributed data$\left({{\varvec{x}}\boldsymbol{^{\prime}}}_{1}.{y}_{1}\right)$,…, $\left({{\varvec{x}}\boldsymbol{^{\prime}}}_{n}.{y}_{n}\right)$ by finding function $y({\varvec{x}})$ as sum of weighted kernels with a small error. The terms $\alpha_{k}^{ + }$ and $\alpha_{k}^{ - }$ are the corresponding Lagrange multipliers for the SVR model and $\mathrm{b}$ is a penalty parameter used to suppress the noise and obtain a soft margin to create a more appropriate and accurate model.

5 $$b = \frac{1}{{N_{v} }}\sum\limits_{v \in V} {\left[ {y_{v} - \varepsilon - \sum\limits_{m = 1}^{n} {(a_{m}^{ + } - a_{m}^{ - } )K(x_{v} ,x_{m} )} } \right]} .$$

**Fig. 5:**
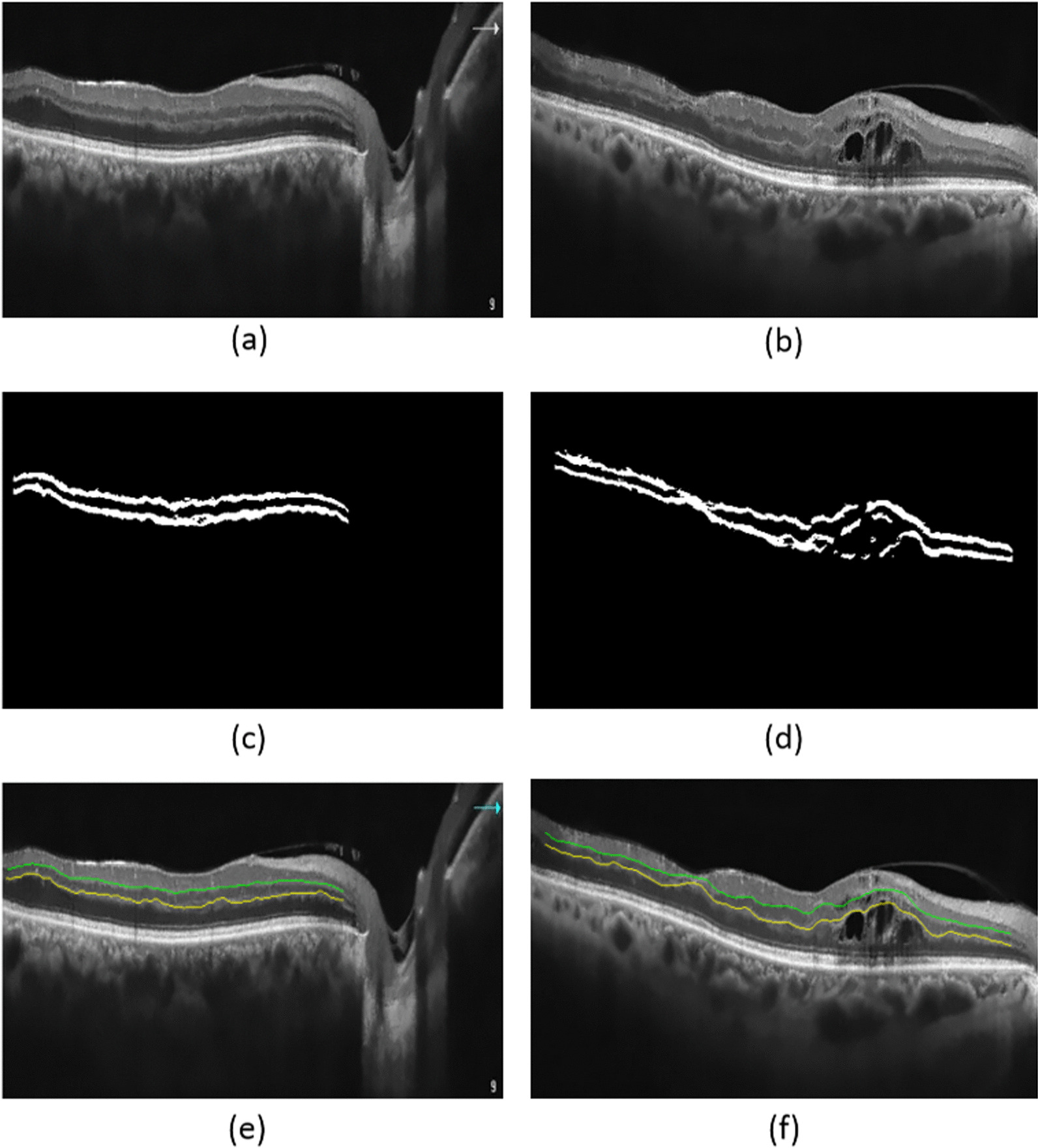
Segmentation using SVR with RBF kernel. **a** Relatively regular layers, **c** The labelling for **a**, **e** Final segmentation for **a**, and **b** Irregular case **d** the labelling IPL and OPL for **b**, **f** Final segmentation of (**b**)

In our work, input $x$ and its corresponding target value $y$ explicitly include the horizontal and vertical coordinates of inner and outer plexiform layer data in the OCT images. Although SVR with RBF kernel is excellent for layer segmentation in noisy images, it performed poorly in OCT slabs with disorganized layers, as demonstrated in Fig. 5d.

Zhang et al., showed that SVR with wavelet kernel has an accurate and acceptable performance against irregularities and fluctuations [47]. The wavelet kernel is a kind of multidimensional wavelet function that can estimate arbitrary nonlinear functions. So kernel function in Eq. (4) should be replaced with Eq. (6) as below:

6 $$K\left( {{\varvec{x}},\user2{x^{\prime}}} \right) = \mathop \prod \limits_{i = 1}^{m} \cos (1.75\frac{{{\varvec{x}}_{{\varvec{i}}} - \user2{x^{\prime}}_{{\varvec{i}}} }}{\sigma })\exp ( - \frac{{{\varvec{x}} - \user2{x^{\prime}}^{2} }}{{2\sigma^{2} }}),$$

With the newly used kernel and fixing the epsilon, penalty parameters and the appropriate choice of $\upsigma$, which is the degree of sensitivity of the kernel to small oscillations, significant results were obtained in dealing with small and large fluctuations in the IPL and OPL layers. To validate the automated algorithm's agreement with manual segmentation of IPL and OPL and also SI measurement in these layers, 50 OCT images with varying levels of retinal layer regularity were chosen (from relatively regular and organized layers in OCT slabs to highly disorganized layers in patients with diabetic retinopathy and diabetic macular edema). Two experts (EKP and HRE) manually segmented IPL and OPL using ImageJ's freehand tool and SI manually computed in each layer of the related OCT slab. The gold standard (GS) for segmentation of the layers and SI calculation was considered by averaging the results of two experts. Additionally, IPL and OPL layers were segmented using the SVR method with a wavelet kernel, and SI was calculated automatically for each layer in all OCT slabs. We utilized the Bland–Altman plot to determine agreement between two experts' manual segmentation and also between the automated method and the manual segmentation.

**Fig. 6:**
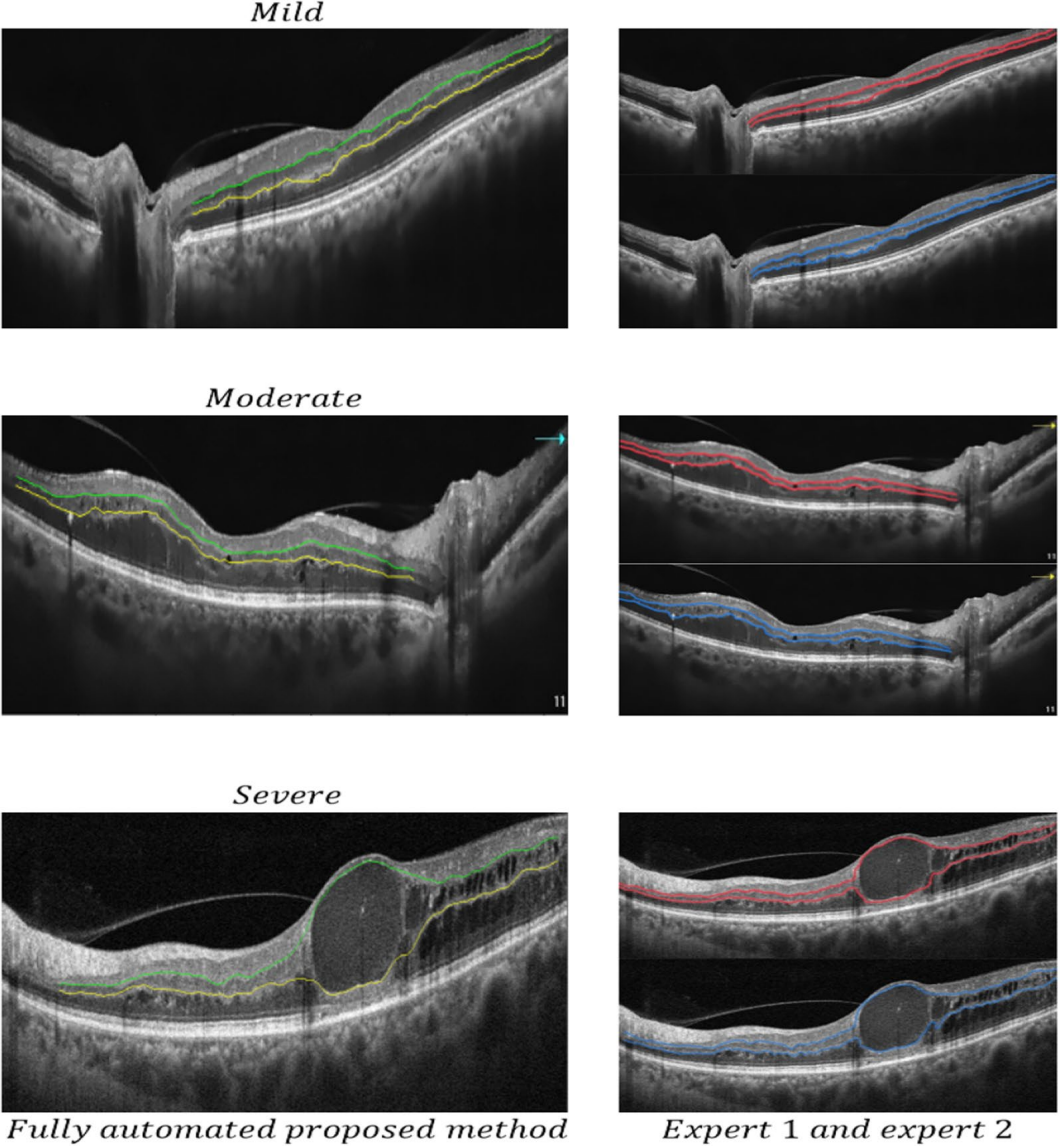
Illustrations of final segmentation results in three different patients with mild, moderate, and severe diabetic retinopathy (rows) with variable irregularity of IPL and OPL by expert 1 (continuous red line), expert 2 (continuous blue line), and fully automated method using SVR with wavelet kernel (continuous green line)

The results of applying the SVR with wavelet kernel and comparison of segmentation with two experts are shown in Fig. 6.

### Quantitative analysis of IPL and OPL layers

**Fig. 7:**
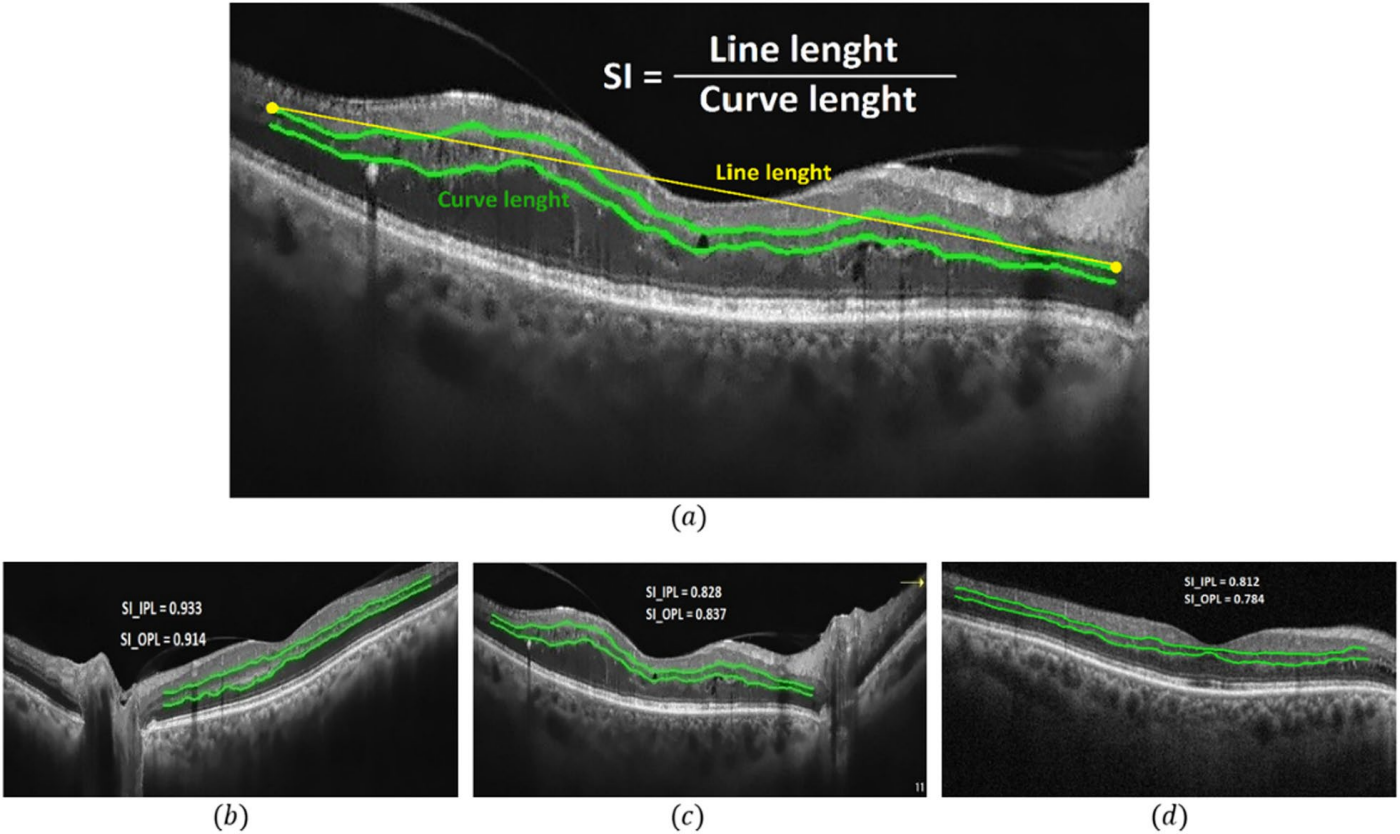
**a** Automated calculation of SI, yellow straight line indicates the Euclidean distance between the beginning and the end point of each layer in the final output image (the length of the straight line connecting the start and the end of IPL or OPL) and, green curves shows the actual length of the layers, obtained by calculating the sum of the Euclidean distance between all the pixels that make up the layer. The results of SI calculation in IPL and OPL for patients with mild, moderate, and severe diabetic retinopathy are depicted in **b**, **c** and **d** respectively

To analysis the IPL and OPL quantitatively, we introduced smoothness index (SI) that can be used to evaluate abnormalities in these layers under pathologic conditions like AMD, Epiretinal Membrane (ERM), and Diabetic Macular Edema (DME) [17–19, 27, 29]. SI was used to evaluate the quantitative smoothness of IPL and OPL layers in macular OCT images and is defined as follows:

7 $$Smoothness \, Index\;(SI) = \frac{{L_{E} }}{{L_{A} }}$$

where ${\mathrm{L}}_{\mathrm{E}}$ indicates the Euclidean distance between the beginning and the end of each layer in the final output image (the length of the straight line connecting the start and the end of IPL or OPL) and, ${\mathrm{L}}_{\mathrm{A}}$ is the actual length of the layer, obtained by calculating the sum of the Euclidean distance between all the pixels that make up the layer (Fig. 7).

## Results

In this study, 50 OCT B-scans from 21 patients with varying stages of diabetic retinopathy were analyzed and two retina experts (EKH and HRE) rechecked and confirmed accurate automated segmentations of IPL and OPL. As previously stated, these 21 patients had varying stages of diabetic retinopathy, with 8 having mild, 9 having moderate, and 4 having severe diabetic retinopathy. Those with mild to moderate diabetic retinopathy have parallel IPL and OPL, whereas patients with severe diabetic retinopathy have disorganized and non-parallel OPL and IPL. We used 42 foveal OCT rasters from 21 patients' both eyes. Meanwhile, we added 8 OCT rasters with disorganized IPL and OPL from the superior or inferior foveal regions of four patients with severe diabetic retinopathy to test the accuracy of the algorithm in segmenting IPL and OPL in circumstances of unorganized OCT images.

The participants' mean age was 61.8 years (range: 47–78 years), and 11 (52.4 percent) were male.

The gold standard (GS), was obtained individually by averaging the layer points marked by two experts. in all of 50 selected OCT slabs with variable fluctuation of layers. IPL and OPL layers were segmented using the SVR method with a wavelet kernel, and SI was calculated automatically for each layer in all 50 OCT slabs. We utilized the Bland–Altman plot to determine agreement between two experts' manual segmentation and automated method. Additional file 1 illustrates the segmentation of all 50 OCT images by two experts and also by automated technique. For validation, the mean unsigned error border position and SI values for each layer were computed and presented in Table 2. The evaluation algorithm is used to test on 50 B-scans. The root mean square error (RMSE) for layer border detection is calculated by Eq. (8), mean absolute error (MAE) is calculated by Eq. (9) and signed error (SE) is calculated by Eq. (10):

8 $$RMSE = \sqrt {\frac{{\sum\nolimits_{{w_{1} }}^{{w_{2} }} {(x_{i} - y_{i} )^{2} } }}{{abs(w_{2} - w_{1} )}},}$$

9 $$MAE = \frac{{\sum\nolimits_{{w_{1} }}^{{w_{2} }} {abs(x_{i} - y_{i} )} }}{{abs(w_{2} - w_{1} )}},$$

10 $$SE = \frac{{\sum\nolimits_{{w_{1} }}^{{w_{2} }} {(x_{i} - y_{i} )} }}{{abs(w_{2} - w_{1} )}},$$

where ${\mathrm{w}}_{1}$ and ${\mathrm{w}}_{2}$ are respectively the common starting point (The first pixel to be marked as the start of the layer in both automatic and GS and the common end point in the automatic methods and GS in each layer. ${\mathrm{x}}_{\mathrm{i}}$, ${\mathrm{y}}_{\mathrm{i}}$ represent the $\mathrm{i}$ th point of the two acquired boundaries for automatic method and GS.

**Table 2:** MSE, MAE and signed error border position (in pixels) between our proposed fully-automated method and the GS

	RMSE		MAE		SE	
	IPL	OPL	IPL	OPL	IPL	OPL
Between two experts	1.24	1.42	0.2	0.25	− 0.013	0.027
Between proposed method and GS	1.79	1.89	0.37	0.49	0.03	− 0.042

Also, the MAE for SI is calculated by:

11 $$MAE = \frac{{\sum\nolimits_{i = 1}^{N} {abs(x_{i} - y_{i} )} }}{N},$$

where $\mathrm{N}$ is number of our 50 B-scans used for test and ${\mathrm{x}}_{\mathrm{i}}$, ${\mathrm{y}}_{\mathrm{i}}$ represent the $\mathrm{i}$ th SI of the two acquired boundaries calculated for automatic method and GS. The value of MAE calculated for SI, between the proposed method and GS, for the two layers IPL and OPL shows the values of 0.016 pixel and 0.025 pixel, respectively.

**Fig. 8:**
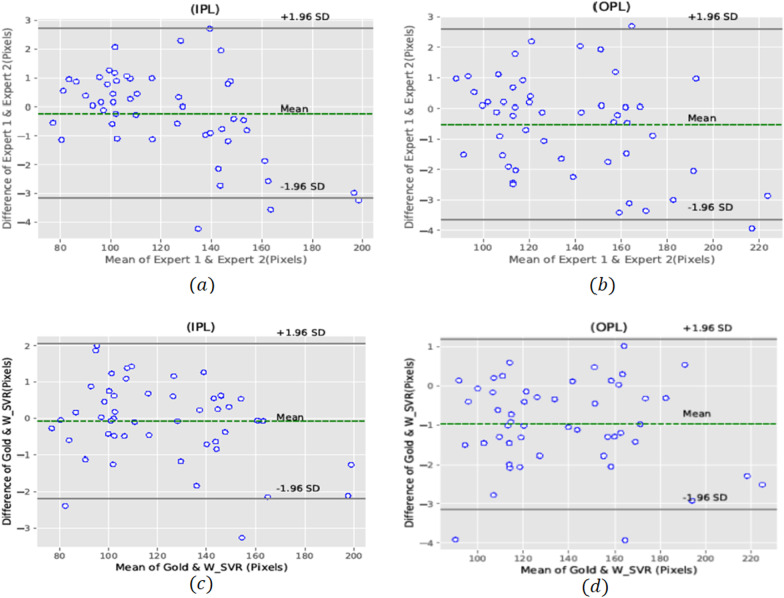
Bland–Altman plots for correlating two experts in manual segmentation of IPL **a** and OPL **b**, also our proposed fully automated algorithm with the GS (average of two experts) for IPL **c** and OPL **d**

Bland–Altman plots for two experts in manual segmentation of IPL and OPL are shown in Fig. 8a–b. As can be seen, acceptable agreement is achieved between two experts for IPL and OPL segmentation. The total agreement between the automated method (SVR with wavelet kernel) and the GS (average of two experts) in IPL and OPL segmentation were also satisfactory, being as shown in Fig. 8c–d. Despite the occurrence of the fluctuations and irregularities in these layers, there was acceptable agreement between the fully-automated method and the GS.

**Fig. 9:**
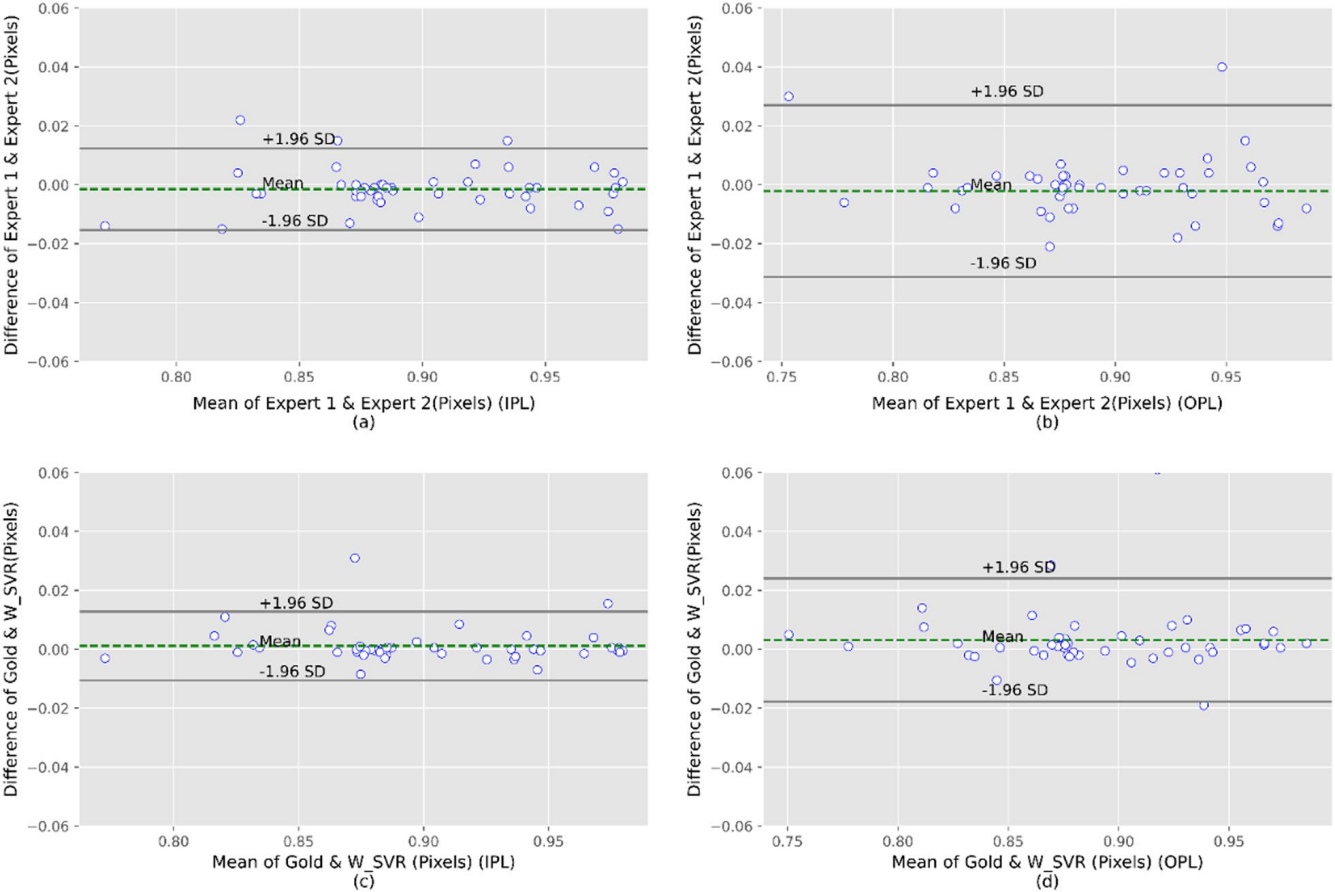
The Bland–Altman plots for the agreement of SI calculation between two experts in IPL (**a**) and OPL (**b**), and also proposed automated method with GS (average of two experts) in SI calculation for IPL (**c**) and OPL (**d**)

Figure 9 depicts the Bland–Altman plots of two experts in manual SI calculation as well as level of agreement between the automated approach and the GS in evaluating the SI measurement. To determine SI value in IPL and OPL layers by two experts, a total agreement of 94 percent is attained. As shown in Fig. 9c–d, there is a high degree of agreement between the automated and manual computation of SI, with 96 and 94 percent agreement in IPL and OPL, respectively.

In general, the Bland–Altman plots showed no substantial difference between manual segmentation technique and our proposed automated approach could be successfully replaced with typical manual segmentation method.

Finally, the execution times for all three methods, including spline interpolation, SVR (RBF kernel), and SVR (Wavelet kernel) are shown in Table 3, which shows that all three perform faster than the manual method for layers’ segmentation.

**Table 3:** A comparison between the required amount of time (in seconds) for all methods and manual method

Layer	Cubic Spline Interpolation	SVR (RBF)	SVR (Wavelet)	manual
IPL	2.5	4.1	4.3	22
OPL	2.7	4.1	4.3	25

## Discussion

In this study, we developed a fully-automated algorithm using machine learning methods to segment the IPL and OPL and calculate the SI in each of these retinal layers. In general, the Bland–Altman plots demonstrated an excellent level of agreement between the two experts and our fully automated method for segmentation and SI calculation in the above-mentioned layers.

We hypothesized that the IPL and OPL would grow increasingly irregular before complete disorganization and the emergence of DRILs. Because of the difficulty in quantifying these abnormalities, prior researches have not looked into the relationship between the irregularity of these layers as a biomarker and visual acuity prognosis or response to therapy. As a result, in order to explore the presence of these connections, an algorithm to quantify the abnormalities of these layers must be designed. We previously incorporated a new biomarker, iris smoothness index (SI), into anterior segment optical coherence tomography (AS-OCT) images of Fuchs uveitis patients and created an automated technique for measuring it [38, 39]. We performed the present research in order to integrate a similar method into retinal layers. The manual segmentation of retinal layers in OCT images using ImageJ software, which is time-consuming and needs a skilled operator, may restrict the practical applicability of SI quantification in retinal layers. To overcome these limitations, the goal of the current study was to create an automated algorithm for calculating the smoothness index in two hyperreflective inner retinal layers (IPL) and (OPL) in patients with varying stages of diabetic retinopathy and diabetic macular edema and compare the results to those obtained using the manual method. In the present research, we attempted to incorporate OCTs with varying degrees of irregularity in order to enhance the algorithm's performance in situations when the presence of macular edema causes severe irregularities in the retinal layers. We enhanced the algorithm's accuracy in IPL and OPL segmentation, as well as SI measurement, by using a machine learning technique called SVR with a wavelet kernel that exhibited consistent agreement with experts and a manual approach.

Figures 4 and 5 show the qualitative representation of IPL and OPL layers segmentation, using classic (cubic spline) and machine learning (SVR) algorithms. Although cubic spline, was a faster way to split layers than other methods (see Table 3), but it failed in OCT images with significant irregularity of layers. SVR with wavelet kernel is shown to be a powerful and robust method for segmentation of the layers with irregularities which could be due to the prediction of the missing values. Also, Fig. 6 depicts an illustration of segmentation results in three different patients (mild, moderate, and severe diabetic retinopathy) with variable irregularity of IPL and OPL, which shows an acceptable agreement between the results obtained by our proposed method and experts' manual segmentation. The comparisons is available in Additional file 1.

Qualitatively, these results in our study are comparable to those of Chiu et al. [48, 49], Dufour et al. [50], Kugelman et al. [51], who implemented and analyzed different automatic segmentation methods to detect retinal layers in optical coherence tomography (OCT) or spectral-domain OCT (SD-OCT) images. Their studies also corroborate this fact that the segmentation challenges increase with the increasing abnormality and irregularity in diabetic retinopathy patients.

Table 2 also shows quantitative results of error measurement for the border position (in pixels) between our proposed fully-automated method and those obtained by experts using the manual method as the ground truth. These measurements were done for three types of error, including root mean square error (RMSE), mean absolute error (MAE), and signed error (SE). Overall, as we can see, low errors were obtained for estimated borders (IPL/OPL) in comparison with the ground truth. RMSE is the standard deviation of the residuals between the estimated and real data. Residuals are a measure of how far from the regression line data points are; RMSE is a measure of how spread out these residuals are. In other words, it tells you how data is concentrated around the line of best fit. In addition, MAE represents the absolute error of our estimation, point by point, and finally, the SE can tell us how well an estimated data matches the quantity that it is supposed to estimate, in which positive and negative errors represent regression situated above and under the line of best fit, respectively.

As a quantitative comparison, these calculations are consistent with the study by Chiu et al. [48, 49], Dufour et al. [50], Kugelman et al. [51], Bilc et al. [52], in which authors provided values of retinal layers segmentation errors obtained from comparison between their automatic algorithm and expert manual segmentation. Also, comprehensive comparisons between these works and our proposed algorithm, including methods, dataset, retinal conditions, retinal layers, segmentation errors, and execution time are listed in Table 4. It can be shown that our observed errors are acceptable and near to the total errors in previous studies, although in our study patients with different stages of diabetic retinopathy were evaluated and might be attributed to the more complicated nature of our segmentation.

**Table 4:** A comprehensive comparison between related works

Year	Study	Dataset	Method	Layers	Error	Time (S)
2010	[48]	Healthy adults	GT + DP	8 boundaries	Layer thickness difference MAE GCL-IPL = 0.77 $\pm$ 0.65 px OPL = 1.48 $\pm$ 1.05 px (px = 3.29 um)	9.74
2013	[50]	Healthy	GT + energy minimization	6 boundaries	MAE IPL-INL = 4.67 $\pm$ 0.83 um SE IPL-INL = − 3.59 $\pm$ 0.93 um (1px = 3.9 um)	18
2015	[49]	Healthy and DME	KR + GT + DP	8 boundaries + fluid	Layer thickness differences GCL-IPL = 4.84 $\pm$ 5.12 um OPL = 6.35 $\pm$ 6.11 um (px = 3.87 um)	11.4
2018	[51]	Healthy children’s and AMD	RNN-GS	7 boundaries of healthy, 3 of AMD	SE INL/IPL = − 0.13 $\pm$ 1.10 px OPL/INL = − 0.10 $\pm$ 1.31 px MAE INL/IPL = 0.56 $\pm$ 0.95 px OPL/INL = 0.69 $\pm$ 1.12 px (px = 3.9 μm)	145
2021	[52]	Chiu dataset, Healthy, AMD, CSCR and DME	GT + WGD	8 boundaries	SE IPL-INL = − 8.12 $\pm$ 5.59 INL-OPL = − 8.33 $\pm$ 7.68 OPL-ONL = − 1.41 $\pm$ 3.51 MAE IPL-INL = 12.67 $\pm$ 3.49 INL-OPL = 7.88 $\pm$ 6.96 OPL-ONL = 1.84 $\pm$ 3.28	–

*GT Graph Theory, *DP* Dynamic Programming, *KR* kernel regression, *WGD* weighted Geodesic Distance, *CSCR* Central Serous Chorioretinopathy

Although, to the best of our knowledge, the measurement of the smoothness (irregularity) of retinal layers has only been done semi-automatically or manually in ERM [30], this potential biomarker of retinal layers in various vascular and structural diseases of the retina can be investigated using the algorithm developed in the current study. In addition to the overall thickness of the retina, which is a popular biomarker, the smoothness index may be used to quantify the response to therapy and prognosis of various patients by conducting longitudinal comparative studies. Although in the current study, SI was calculated in the OCT images of patients with varying stages of diabetic retinopathy, to conclusively determine whether or not this index is a novel biomarker, larger-scale investigations will be required.

While we have developed a relatively reliable and convenient technique for detecting hyperreflective OCT layers and corresponding smoothness index, it should be warned that in certain situations when layer irregularity and oscillation are significant and layers are indistinguishable like in cases of DRIL, this method may not give a correct answer.

**Fig. 10:**
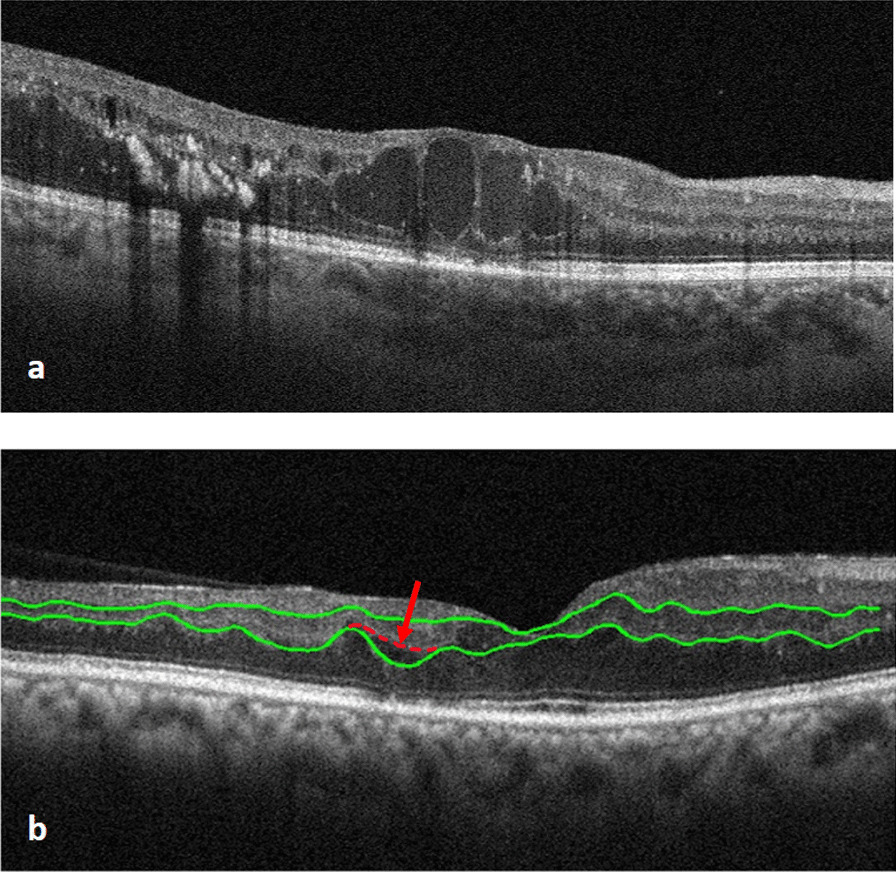
An example of OCT raster was so unstructured that identifying IPL and OPL was impossible even with manual segmentation, therefore they were omitted from the study (**a**). An example of segmentation error in OPL in a case with high level of irregularity and oscillation and poor OPL contrast (**b**)

Although, we added 8 OCT rasters with disorganized IPL and OPL from the superior or inferior foveal regions of four patients with severe diabetic retinopathy to test the accuracy of the algorithm in segmenting IPL and OPL in circumstances of unorganized OCT images. Some of these OCT rasters were so unstructured that identifying IPL and OPL was impossible even with manual segmentation (Fig. 10a), therefore they were omitted from the study. Our implemented algorithm, on the other hand, failed to correctly separate the IPL / OPL in two scans when layer irregularity and oscillation are high and layers are fade due to poor image quality or contrast (Fig. 10b).

On the other hand, we have just recruited patients with diabetic retinopathy in order to evaluate the performance and accuracy of the proposed algorithm. To address this restriction, an investigation of the current algorithm's accuracy on a large sample size of individuals with various retinal diseases should be conducted.

## Conclusion

In conclusion, in the current study we developed a reliable, fast and convenient algorithm based on machine learning method to automatically segment two hyperreflective inner retinal layers and calculate smoothness index (SI) in the corresponding layers. Results prove that there is an acceptable agreement between our proposed algorithm and those obtained by experts using the manual method as the ground truth and therefore, the SI can be used to quantify the response to therapy and prognosis of various patients by conducting longitudinal comparative studies.

## Supplementary Information

**Additional file 1:** Segmentation of the IPL and OPL by two specialists and the automated approach.

## Data Availability

The data that support the findings of this study are available from [Elias Khalili Pour] but restrictions apply to the availability of these data, which were used under license for the current study, and so are not publicly available. Data are however available from the authors upon reasonable request and with permission of [Elias Khalili Pour].

## Abbreviations

OCT: Optical coherence tomography
SD-OCT: Spectral-domain optical coherence tomography
ERM: Epiretinal membranes
IPL: Inner plexiform layer
OPL: Outer plexiform layer
AMD: Age-related macular degeneration
ILM: Internal limiting membrane
RPE: Retinal pigment epithelium
DRIL: Disorganization of the retinal inner layers
SVR: Support vector regression
RBF: Radial basis functions
SI: Smoothness index
RMSE: Root mean square error
MAE: Mean absolute error
SE: Signed error

## Code availability

The machine-learning models were developed and deployed using standard model libraries and the Python framework. The codes may be available for research purposes from the corresponding authors on reasonable request.

## References

[1] Podoleanu AG (2005). Optical coherence tomography. Br J Radiol.

[2] Huang D, Swanson EA, Lin CP, Schuman JS, Stinson WG, Chang W (1991). Optical coherence tomography. Science.

[3] Schuman JS, Hee MR, Arya AV, Pedut-Kloizman T, Puliafito CA, Fujimoto JG (1995). Optical coherence tomography: a new tool for glaucoma diagnosis. Curr Opin Ophthalmol.

[4] Coker JG, Duker JS (1996). Macular disease and optical coherence tomography. Curr Opin Ophthalmol.

[5] Mohammadzadeh V, Fatehi N, Yarmohammadi A, Lee JW, Sharifipour F, Daneshvar R (2020). Macular imaging with optical coherence tomography in glaucoma. Surv Ophthalmol.

[6] Hee MR (1995). Optical coherence tomography of the human retina. Arch Ophthalmol.

[7] Kafieh R, Amini Z, Rabbani H, Baghbaderani BK, Salafian B, Mazaheri F (2019). Automatic Multifaceted matlab package for analysis of ocular images (AMPAO). SoftwareX.

[8] Kafieh R, Rabbani H, Abramoff MD, Sonka M (2013). Intra-retinal layer segmentation of 3D optical coherence tomography using coarse grained diffusion map. Med Image Anal.

[9] Rabbani H, Kafieh R, Kermani S (2013). A review of algorithms for segmentation of optical coherence tomography from retina. J Med Signals Sensors.

[10] González-López A, de Moura J, Novo J, Ortega M, Penedo MG (2019). Robust segmentation of retinal layers in optical coherence tomography images based on a multistage active contour model. Heliyon.

[11] Montazerin M, Sajjadifar Z, Pour EK, Riazi-Esfahani H, Mahmoudi T, Rabbani H (2021). Livelayer: a semi-automatic software program for segmentation of layers and diabetic macular edema in optical coherence tomography images. Sci Rep.

[12] Alasil T, Keane PA, Updike JF, Dustin L, Ouyang Y, Walsh AC (2010). Relationship between optical coherence tomography retinal parameters and visual acuity in diabetic macular edema. Ophthalmology.

[13] Uji A, Murakami T, Nishijima K, Akagi T, Horii T, Arakawa N (2012). Association between hyperreflective foci in the outer retina, status of photoreceptor layer, and visual acuity in diabetic macular edema. Am J Ophthalmol.

[14] Lai TY, Saxena S, Srivastav K, Cheung G, Ng YWJ. Photoreceptor inner segment ellipsoid band integrity on spectral domain optical coherence tomography. Clin Ophthalmol. 2014:2507. 10.2147/OPTH.S72132 PMC4266419 25525329

[15] Chan EW, Eldeeb M, Sun V, Thomas D, Omar A, Kapusta MA (2019). Disorganization of retinal inner layers and ellipsoid zone disruption predict visual outcomes in central retinal vein occlusion. Ophthalmol Retin.

[16] Wu SM (2010). Synaptic organization of the vertebrate retina: general principles and species-specific variations: the Friedenwald lecture. Invest Ophthalmol Vis Sci.

[17] Sun JK, Lin MM, Lammer J, Prager S, Sarangi R, Silva PS (2014). Disorganization of the retinal inner layers as a predictor of visual acuity in eyes with center-involved diabetic macular edema. JAMA Ophthalmol.

[18] Goker YS, Atılgan CU, Tekin K, Kızıltoprak H, Kosekahya P, Demir G (2020). Association between disorganization of the retinal inner layers and capillary nonperfusion area in patients with retinal vein occlusion. Arq Bras Oftalmol.

[19] Das R, Spence G, Hogg RE, Stevenson M, Chakravarthy U (2018). Disorganization of inner retina and outer retinal morphology in diabetic macular edema. JAMA Ophthalmol.

[20] Garnavou-Xirou C, Xirou T, Gkizis I, Kabanarou SA, Dimitriou E, Theodossiadis P (2019). The role of disorganization of retinal inner layers as predictive factor of postoperative outcome in patients with epiretinal membrane. Ophthalmic Res.

[21] Disorganization of the retinal inner layers as a prognostic factor in eyes with central retinal artery occlusion. Int J Ophthalmol. 2019;12. 10.18240/ijo.2019.06.18 PMC6580216 31236358

[22] Grewal DS, O’Sullivan ML, Kron M, Jaffe GJ (2017). Association of disorganization of retinal inner layers with visual acuity in eyes with uveitic cystoid macular edema. Am J Ophthalmol.

[23] Călugăru D, Călugăru M (2017). Disorganization of the retinal inner layers as a predictor of visual acuity in eyes with macular edema secondary to vein occlusion. Am J Ophthalmol.

[24] Joltikov KA, Sesi CA, de Castro VM, Davila JR, Anand R, Khan SM (2018). Disorganization of retinal inner layers (DRIL) and neuroretinal dysfunction in early diabetic retinopathy. Investig Opthalmology Vis Sci.

[25] Nadri G, Saxena S, Stefanickova J, Ziak P, Benacka J, Gilhotra JS (2019). Disorganization of retinal inner layers correlates with ellipsoid zone disruption and retinal nerve fiber layer thinning in diabetic retinopathy. J Diabetes Complicat.

[26] Guo J, Tang W, Ye X, Wu H, Xu G, Liu W, et al. Predictive multi-imaging biomarkers relevant for visual acuity in idiopathic macular telangiectasis type 1. BMC Ophthalmol. 2018;18. 10.1186/s12886-018-0737-y PMC5836363 29506510

[27] Zur D, Iglicki M, Feldinger L, Schwartz S, Goldstein M, Loewenstein A (2018). Disorganization of retinal inner layers as a biomarker for idiopathic epiretinal membrane after macular surgery-the DREAM study. Am J Ophthalmol.

[28] Ishibashi T, Sakimoto S, Shiraki N, Nishida K, Sakaguchi H, Nishida K (2019). Association between disorganization of retinal inner layers and visual acuity after proliferative diabetic retinopathy surgery. Sci Rep.

[29] Schmidt-Erfurth U, Michl M (2019). Disorganization of retinal inner layers and the importance of setting boundaries. JAMA Ophthalmol.

[30] Cho KH, Park SJ, Cho JH, Woo SJ, Park KH (2016). Inner-retinal irregularity index predicts postoperative visual prognosis in idiopathic epiretinal membrane. Am J Ophthalmol.

[31] Mishra Z, Ganegoda A, Selicha J, Wang Z, Sadda SR, Hu Z (2020). Automated retinal layer segmentation using graph-based algorithm incorporating deep-learning-derived information. Sci Rep.

[32] He Y, Carass A, Liu Y, Jedynak BM, Solomon SD, Saidha S (2019). Fully convolutional boundary regression for retina OCT segmentation. Med image Comput Comput Interv MICCAI Int Conf Med Image Comput Comput Interv.

[33] Huang Y, Danis RP, Pak JW, Luo S, White J, Zhang X (2013). Development of a semi-automatic segmentation method for retinal OCT images tested in patients with diabetic macular edema. PLoS ONE.

[34] Liu X, Fu T, Pan Z, Liu D, Hu W, Li B. Semi-supervised automatic layer and fluid region segmentation of retinal optical coherence tomography images using adversarial learning. In: 2018 25th IEEE International Conference on Image Processing (ICIP). 2018.

[35] Koozekanani D, Boyer K, Roberts C (2001). Retinal thickness measurements from optical coherence tomography using a Markov boundary model. IEEE Trans Med Imaging.

[36] Munk MR, Beck M, Kolb S, Larsen M, Hamann S, Valmaggia C (2017). Quantification of retinal layer thickness changes in acute macular neuroretinopathy. Br J Ophthalmol.

[37] Mariottoni EB, Jammal AA, Urata CN, Berchuck SI, Thompson AC, Estrela T (2020). Quantification of retinal nerve fibre layer thickness on optical coherence tomography with a deep learning segmentation-free approach. Sci Rep.

[38] Zarei M, Mahmoudi T, Riazi-Esfahani H, Mousavi B, Ebrahimiadib N, Yaseri M (2021). Automated measurement of iris surface smoothness using anterior segment optical coherence tomography. Sci Rep.

[39] Zarei M, KhaliliPour E, Ebrahimiadib N, Riazi-Esfahani H. Quantitative analysis of the iris surface smoothness by anterior segment optical coherence tomography in Fuchs Uveitis. Ocul Immunol Inflamm. 2020;1–6. 10.1080/09273948.2020.1823424 32966151

[40] Sartor LJ, Weeks AR (2001). Morphological operations on color images. J Electron Imaging.

[41] Schoenberg IJ. Cardinal spline interpolation. SIAM. 1973.

[42] Cristianini N, Shawe-Taylor J. An introduction to support vector machines and other kernel-based learning methods. Cambridge university press; 2000.

[43] Zhou D-X, Jetter K (2006). Approximation with polynomial kernels and SVM classifiers. Adv Comput Math.

[44] Lin H-T. A study on sigmoid kernels for SVM and the training of non-PSD kernels by SMO-type methods. 2005.

[45] Xiaoyan WPZ (2003). Model selection of SVM with RBF kernel and its application. Comput Eng Appl.

[46] Steinwart I, Hush D, Scovel C (2006). An explicit description of the reproducing kernel Hilbert spaces of Gaussian RBF kernels. IEEE Trans Inf Theory.

[47] Zhang L, Zhou W, Jiao L (2004). Wavelet support vector machine. IEEE Trans Syst Man Cybern Part B.

[48] Chiu SJ, Li XT, Nicholas P, Toth CA, Izatt JA, Farsiu S (2010). Automatic segmentation of seven retinal layers in SDOCT images congruent with expert manual segmentation. Opt Express.

[49] Chiu SJ, Allingham MJ, Mettu PS, Cousins SW, Izatt JA, Farsiu S (2015). Kernel regression based segmentation of optical coherence tomography images with diabetic macular edema. Biomed Opt Express.

[50] Dufour PA, Ceklic L, Abdillahi H, Schroder S, De Dzanet S, Wolf-Schnurrbusch U (2013). Graph-based multi-surface segmentation of OCT data using trained hard and soft constraints. IEEE Trans Med Imaging.

[51] Kugelman J, Alonso-Caneiro D, Read SA, Vincent SJ, Collins MJ (2018). Automatic segmentation of OCT retinal boundaries using recurrent neural networks and graph search. Biomed Opt Express.

[52] Bilc S, Groza A, Muntean G, Nicoara SD (2021). Interleaving automatic segmentation and expert opinion for retinal conditions. Diagnostics.

